# Venous blood point-of-care testing (POCT) for paramedics in urgent and emergency care: a single-site feasibility study (POCTPara)

**DOI:** 10.29045/14784726.2025.6.10.1.19

**Published:** 2025-06-01

**Authors:** Andrew Hodge, Bryan Lightowler, Richard Pilbery, Fiona Bell, Pete Best, Kelly Hird, Beverly Snaith, Alison Walker

**Affiliations:** North East Ambulance Service NHS Foundation Trust ORCID iD: https://orcid/org/0000-0002-2632-2249; University of Bradford ORCID iD: https://orcid/org/0000-0002-9884-6762; Yorkshire Ambulance Service NHS Trust ORCID iD: https://orcid.org/0000-0002-5797-9788; Yorkshire Ambulance Service NHS Trust ORCID iD: https://orcid.org/0000-0003-4503-1903; Calderdale and Huddersfield NHS Foundation Trust ORCID iD: https://orcid.org/0009-0002-7298-1138; Yorkshire Ambulance Service NHS Trust ORCID iD: https://orcid.org/0009-0000-1146-022X; Mid Yorkshire Teaching NHS Trust; University of Bradford ORCID iD: https://orcid.org/0000-0002-6296-0889; Harrogate and District NHS Foundation Trust ORCID iD: https://orcid.org/0009-0000-9235-2676

**Keywords:** paramedics, point-of-care test, pre-hospital care

## Abstract

**Introduction:**

Paramedics play an important role in addressing the growing demands in urgent and emergency care. Point-of-care testing (POCT) devices are increasingly portable and may assist with appropriate non-conveyance, but limited research exists to support this. This feasibility study aimed to inform the design of a larger study to determine whether it is practical for paramedics to use blood analysis POCT.

**Methods:**

An eight-month single-site feasibility sequential explanatory mixed-method study was conducted between April and December 2023, with a team of specialist paramedics who were provided with Abbott® *i-STAT Alinity*™ POCT devices with CHEM8+ and CG4+ cartridges. Using a qualitative evaluation of paramedic participants’ experience with a POCT device and a descriptive analysis of case report forms and routine ambulance service data collection.

**Results:**

Seven specialist paramedics were recruited; 287 patients were screened, of which 252 (88%) were excluded and 35 (12%) were recruited. Lack of mental capacity excluded 76% of cases. The mean age was 82 years; 40% of participants were female and 60% were male. Hospital conveyance rates were four (11%) of the recruited patients. In those recruited and not conveyed, the median time on scene was 120 minutes. The success rate to obtain a test result at the first attempt was 81% (CHEM8+) and 84% (CG4+). Test result failure rates were 13% (CHEM8+) and 3% (CG4+). Focus group data revealed that paramedic participants considered POCT useful for decision making and the device procedures to be acceptable. Paramedics reported that extended time on scene was related to trial procedures and waiting times to discuss test results with healthcare professionals.

**Conclusion:**

The POCT devices were acceptable and practical for use by our specialist paramedic participants. The results of this feasibility study should inform the design of a larger study to test the impact of using POCT, to understand challenges in recruitment and retention where POCT is utilised and to determine the clinical presentations where POCT can be applied.

## Introduction

The traditional role of the ambulance service paramedic has expanded from emergency care and transport to include complex, lower-acuity management in the community. The demand for urgent and emergency care services in the NHS continues to increase, with calls for primary care, ambulance services and emergency departments (EDs) increasing (National Audit Office, 2023). Pressures in the health and social care system have been unprecedented since the COVID-19 pandemic, with an association between time spent in ED and mortality, and harm related to ambulance‒hospital handover delays documented (Association of Ambulance Chief Executives, 2012; Office for National Statistics, 2023).

With a national average monthly conveyance rate to the ED of approximately 51.2% (National Audit Office, 2023), there is increasing focus on the role of ambulance services in appropriate admission avoidance as one way to manage the pressure of demand on EDs and hospitals. Studies have demonstrated that paramedics with additional practitioner training can have a positive effect on the management of patients in the community, with NICE guidance making the recommendation to develop these roles as an alternative to ED attendance (Mason et al., 2007, 2012; NICE, 2018).

To address the rise in service demand, technology has been identified as a key enabler for improving joined-up care across systems. Remote diagnostics, such as point-of-care testing (POCT), are seen as one way to aid decision making and thereby improve the efficiency of NHS services and resources (NHS England, 2018). POCT is defined as a near-patient diagnostic investigation outside of the clinical laboratory (Florkowski et al., 2017; International Organization for Standardization, 2019; Larsson et al., 2015), and increasingly portable POCT devices have introduced the capacity for blood test analysis in the community across a range of clinical presentations (Galvagno et al., 2020; Novak et al., 2021). The adoption of POCT in pre-hospital care has largely been restricted to critical and acute care (Stengaard et al., 2013; Stopyra et al., 2020; van Dongen et al., 2018; Younger et al., 2014), but beyond this, there is less evidence related to its application in the more common patient presentations managed by ambulance services (Heaney et al., 2019; Kim & Kim, 2018).

In one small English study, paramedics self-reported improved decision making in 78 cases but highlighted issues surrounding equipment storage, training, quality control and failure rates (McPherson, 2018). Another study involving Canadian community paramedics reported clinically valid results compared with laboratory test results but again reported challenges in terms of device failure rates (Blanchard et al., 2019). A more recent service evaluation suggests that POCT use by paramedics assessing frail patients is a feasible model when combined with a conference call with a senior hospital physician (Novak et al., 2021).

This feasibility study aimed to understand whether it is practical for specialist paramedics to use POCT in the pre-hospital setting for patients with lower acuity presentations. It further aimed to potentially inform the design of a larger future study.

## Methods

The primary objective of the study was to understand the experiences of specialist paramedics using POCT devices in pre-hospital urgent and emergency care settings, along with their self-reported impact on clinical decision making. The secondary objectives were to establish the practicality of using POCT devices in this setting and to understand in which patient presentations the device is most used. Table 1 details the primary and secondary objectives and outcome measures.

**Table 1.** Study objectives and outcome measures.

	Objectives	Outcome measures
**Primary**	To understand the experiences and self-reported impact of using POCT devices on clinical decision making for safely managing patients in the community.	Qualitative focus-group data measuring perceived self-reported impact.
**Secondary**	To understand the practicality of utilising the POCT analyser in the urgent and emergency care setting. To understand the patient populations that the POCT devices are most commonly applied to.	Number and type of POCT cartridges used. Number of successful and unsuccessful attempts in using the POCT device. Length of time on scene. Specialist paramedic recruitment and retention. Number of patients who receive POCT. Descriptive data of safe conveyance (proportion of cases conveyed from overall cases managed) and 72-hour recontact rates (proportion of cases that recontact 999 from overall cases managed). Patient demographics and presentations where POCT is applied – age, sex, NEWS2 score, clinical condition. Data quality.

NEWS2: National Early Warning Score; POCT: point-of-care testing.

### Study design

This research was an eight-month, single-site, feasibility sequential explanatory mixed-method study comprising descriptive data related to the care episode, POCT device use and paramedic time on scene and a qualitative evaluation of the specialist paramedic participants’ experience using POCT devices through a 1.5-hour semi-structured interview focus group.

### Study setting

The study took place in a city in northern England with a population of approximately 550,000 (Office for National Statistics, 2021), where a team of 14 specialist paramedics in urgent care work are based. These clinicians have completed postgraduate MSc-level education that has extended their scope of practice to manage urgent illness and injury presentations in addition to their usual paramedic skills; however, they typically do not have prior experience with POCT. Members of this team are deployed to emergency calls in solo response cars, where they may be able to avoid an ED attendance by organising direct admission or a community care referral or by treating and discharging the patient on scene. Furthermore, they also receive direct referrals from care homes or general practitioners (GPs) for minor injuries, and from local ambulance crews to avoid a hospital attendance where appropriate.

### Participants and recruitment

All 14 specialist paramedics were invited to participate in this study by email; seven were recruited, and their consent was obtained. Training comprised completion of the National Institute for Health and Care Research (NIHR) Good Clinical Practice online training and a seven-hour face-to-face session that covered eligibility assessment, consent and recruitment procedures, test performance, result interpretation, data collection and adverse event (AE) or serious adverse event (SAE) reporting. Ongoing support was available from an advanced paramedic with prior POCT experience in an ED setting.

The study aimed to recruit 50 patients over a six-month period, which was extended to eight months due to recruitment challenges. If eligible for recruitment (Supplementary 1), the specialist paramedic gave the patient scripted verbal information, accompanied by an information leaflet, before completing the consent process if patient agreement was obtained. Owing to the nature of the care setting, any patient who required emergency intervention and transport was not included, and all patients were able to withdraw their consent in the following seven days.

### Trial intervention

Two Abbott i-STAT Alinity POCT devices were used by the specialist paramedics. Quality assurance procedures, involving daily fridge temperature checks for cartridge storage, were required to maintain the devices.

If a patient consented to participate, the specialist paramedics obtained a venous blood sample via a butterfly needle or cannula and a 1 ml heparinised syringe before it was applied to the test cartridge. Owing to time and resource restrictions associated with training and safe use in this feasibility study, only CG4+ and CHEM8+ cartridges were used, and the results were restricted to lactate, sodium, potassium, urea, creatinine and haemoglobin. Both cartridges required a minimum blood sample of 95 μL and provided analysis of the results within 120 seconds.

A quick reference guide to test results with clinical management options was developed and provided to the specialist paramedics, who were also required to discuss the clinical presentation with the patient’s GP or other relevant healthcare professional where there was clinical uncertainty or abnormal results.

### Data collection and analysis

The study data included variables obtained from the ambulance service electronic patient care records and the computer-aided dispatch system. In addition, a study-specific electronic case report form (eCRF) collected additional descriptive data aligned with the secondary outcome measures. Following patient recruitment and the seven-day withdrawal period, all the data were anonymised for analysis.

After the patient recruitment phase, qualitative data were collected through a 1.5-hour semi-structured interview focus group with the specialist paramedics to understand their experiences and the self-reported impact of using the POCT devices. Owing to funding limitations for this feasibility study, the focus group interviews were not recorded and transcribed, but field notes were taken, reviewed independently and organised into common themes by two interviewers (AH and BL, who are both paramedics by profession and knew the paramedic participants from previous employment). The consolidated criteria for reporting qualitative research (COREQ) checklist was considered during the study period (Tong et al., 2007). Supplementary 2 details the focus group interview schedule.

## Results

### Participant characteristics

During the study, the specialist paramedic participant numbers decreased, as two left the organisation and one did not engage further with the study. Among the four remaining participants, all had over 10 years’ experience as paramedics and held at least a postgraduate diploma level 7 qualification, with all the characteristics presented in Table 2.

**Table 2.** Specialist paramedic participant characteristics.

Characteristic	Value category (frequency)	
Age	40–44 (1)	45–49 (3)
Sex	Male (3)	Female (1)
Paramedic experience in years	10–14 (3)	15–20 (1)
Specialist paramedic experience in years	0–5 (2)	6–10 (2)

### Descriptive data

**Figure 1:**
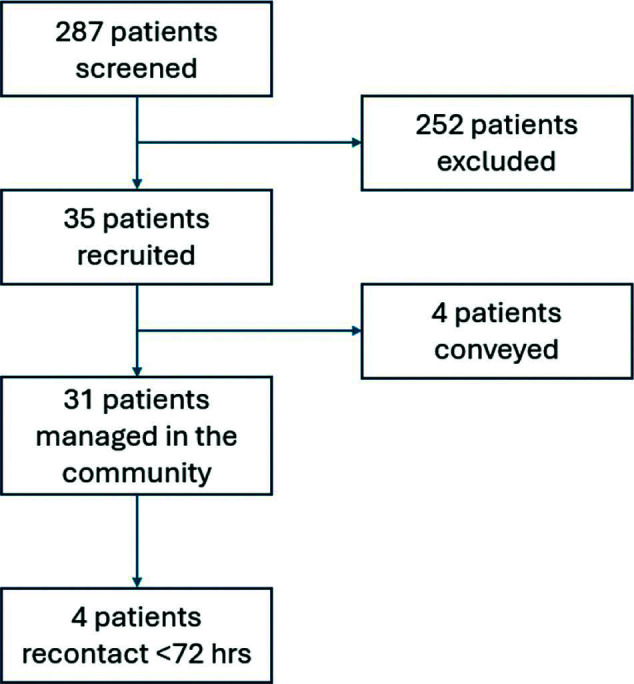
Figure 1. Recruitment flow chart.

During the study period, April to December 2023, the paramedics attended 2253 cases. A total of 287 patients were screened for eligibility, with 252 (88%) excluded and 35 (12%) recruited (Figure 1). Lack of mental capacity was the most common reason (76%) for not meeting the inclusion criteria. The median age of those recruited was 82 years; 14 were female (40%) and 21 were male (60%). The first mean National Early Warning Score (NEWS2) calculated for the recruited patients was 1 (range 0–2), and the mean score was 1 (range 0–3) for those excluded from the study.

Of the 35 recruited patients, four (11%) were conveyed to hospital (2 = ED; 2 = ambulatory care unit). Of those not conveyed, the median time on scene was 120 minutes (range 99–144) for those recruited. Table 3 presents the clinical impressions recorded by the specialist paramedics, with falls accounting for nearly half of those patients who received POCT. Among the 287 patients screened in this study, four of the 35 recruited patients (11%) recontacted the ambulance service within 72 hours. There were no cases of harm reported using protocol criteria.

**Table 3.** Clinical impressions where POCT was applied.

Condition	Number (%)
Fall (intrinsic/extrinsic)	16 (46)
LRTI / exacerbation COPD	5 (14)
Urinary tract infection	4 (11)
Syncope/vasovagal	3 (8)
Other infection (e.g. cellulitis, septic arthritis)	2 (6)
Asthma	1 (3)
Abdominal pain / nausea	1 (3)
Jaundice	1 (3)
Generalised pain	1 (3)
Post-hospital admission recovery	1 (3)

Of the 35 patients recruited for the study, 32 had a POCT conducted. The reasons for not completing the POC test were inability to obtain a venous blood sample (n = 1), not attempting a venous blood draw (n = 1) or failure of the device to register the cartridge (n = 1).

Table 4 shows the types of cartridges used, with an 81% success rate at the first attempt for the CHEM8+ cartridge and an 84% success rate at the first attempt for the CG4+ cartridge. The failure rate to obtain a test result with the CHEM8+ cartridge was 13% (n = 4), and with the CG4+ cartridge was 3% (n = 1). The failure of the CHEM8+ cartridge was most often related to cartridge quality issues, and the failure of the CG4+ cartridge was most often due to user error related to cartridge blood fill volumes.

**Table 4.** Cartridge types used, success rates and failure reasons.

Cartridge type / number of attempts	Fill success (cartridges used)	Failure rate reasons
CHEM8+		Damaged barcode, failed quality-check code, cartridge failure (32-01), cartridge quality error, cartridge registering out of date, cartridge overfilled.
1st attempt success	26 (26)	
2nd attempt success	2 (4)	
Aborted	4 (5)	
CG4+		
1st attempt success	27 (27)	
2nd attempt success	3 (6)	Cartridge low-fill code (23-01), cartridge too little / too much blood code, cartridge overfilled x2.
3rd attempt success	1 (3)	
Aborted	1 (1)	

### Qualitative data

Four of the seven specialist paramedic participants initially recruited for the study took part in the focus-group interview. In accordance with the descriptive data, there was a consensus among the interview participants that on-scene times significantly increased when using POCT, which was associated with recruitment trial procedures and waiting for GP callbacks to discuss the case and test results. Challenges with recruiting patients who met the eligibility criteria were thought to be caused by several factors, with the exclusion criteria of lack of mental capacity ruling out most patients, but also the participants reported being dispatched to higher acuity emergency calls, limiting exposure to appropriate cases. During the trial period, some of the paramedic participants reported that they began rotational work through primary care placements in six-week blocks, further reducing opportunities for pre-hospital patient recruitment.

Quality control procedures were generally acceptable, with fridge storage and temperature monitoring processes manageable; however, caution was needed, as the expiry date was printed on the cartridge in the American format (month/date/year), introducing the potential for confusion, with one participant citing this as a reason for one instance of device setup failure and cartridge waste.

Device use was reported to be intuitive once practice had helped to overcome any challenges associated with a lack of familiarity. Cartridge overfilling was one of the most common reasons for failure, and this was reported to improve with time after participants realised that empty space was needed in the cartridge for the closure port to close. Another issue related to the colour of the cartridges, which differed from the colour of their packaging, and resulted in one participant opening and spoiling the wrong cartridge. Device failures associated with operating temperatures beyond the reference range were not reported, although one case was described where the cartridge needed to come back up to room temperature before use.

There was consensus among the specialist paramedic participants that POCT devices have utility in the pre-hospital environment and that they benefit decision making to support appropriate management. Training was considered key to successful implementation of the devices, with more education essential if independent test result interpretation was needed. Moreover, a community of practice for peer support was suggested for those using POCT in the pre-hospital environment if this was to become more widely adopted and embedded into practice.

While the group felt that POCT offered benefits in decision making, particularly in cases of infection, patients with long periods of immobility after a fall on the floor and those with non-specific symptoms, they also felt that it could improve antimicrobial stewardship but emphasised the need to avoid taking on the work of other services.

## Discussion

To our knowledge, this feasibility study is one of the few published that relates to the practical use of POCT blood analysis by paramedics in the pre-hospital setting for lower acuity patients.

A key finding in this feasibility study was that by omitting patients without mental capacity, 76% of the patients screened were excluded. In their study, Blanchard et al. (2019) also excluded patients without capacity and did not apply POCT devices in 93.5% of their total patient episodes screened, although the reasons for this were not reported. Additionally, Heaney et al. (2019) used POCT in 25% of patient interactions, although as a service evaluation there were no research recruitment or eligibility decisions. For our small-scale feasibility study, the exclusion of patients without capacity was a pragmatic decision taken to evaluate the experience and potential of paramedics to utilise the technology in practice. This decision also likely influenced the low screening and recruitment number in comparison to the number of patient episodes during the study period. For future research, the implications of patient capacity would have to be considered with inclusion and exclusion criteria appropriate for the population and the research question.

Another key finding suggests that POCT devices have the potential to significantly extend the time spent on scene with the patient, with our data suggesting that this could be doubled compared with usual care. However, our paramedic participants reported that this was influenced by trial recruitment procedures and the need to wait to speak to the patient’s GP, rather than it being related to the test process. Future studies could ensure that consent procedures are more straightforward and that further education and senior clinical advice, either by peer support or from within the ambulance setting, are provided to increase interpretation confidence. Furthermore, it should also be considered whether extended time at the scene is still better for both patient care and healthcare resource use than extended waiting times at hospitals for handover and the risks associated with hospital admissions.

Our findings of an 81–84% success rate at the first attempt to obtain a test result suggest that paramedics can practically use the device. This finding correlates with our qualitative findings that reported relative ease of use after initial challenges related to familiarisation, which is important given the cost of cartridges. Some of these test failures were related to overfilling cartridges, which correlates with the findings of Blanchard et al. (2019), where most failure reasons were human rather than equipment errors. Failure reasons should be analysed and used to inform education programmes aimed at minimising waste; however, device manufacturers should also consider user feedback, e.g. regarding cartridge colour packaging and American date formats which may cause confusion and increase the costs associated with wasted cartridges.

We restricted the range of tests available for use to make the training practical and achievable. As a result, the clinical presentations to which the devices were applied may not reflect the potential range of use in the pre-hospital urgent and emergency care setting. Our findings show that falls accounted for nearly half of all presentations. Further research should consider the application of testing a population where the most benefit from community management could be achieved, such as falls in older adults where capacity may be impaired.

When tests are carried out, our data suggest that POCT may have the potential to improve decision making on community management rather than conveyance to hospital without compromising patient safety, as both recontact rates and adverse events were low, which is similar to other studies (Heaney et al., 2019; Novak et al., 2021). While our feasibility study indicates that the use of POCT by specialist paramedics is acceptable, the paramedic participants recruited for this study may have biased this view, as they were willing to participate and provided data on their experiences with device use. However, other members of the team who were invited to participate did not do so, and future research may need to understand this as potential barriers to device utilisation.

Since paramedics are not traditionally familiar with POCT, key factors for successful implementation were education, clinical supervision and the existence of a community of practice. Further work is required to understand this, as ongoing device utilisation must be sustainable for patient and system benefits to be realised. One key separate development that occurred during this study was the introduction of primary care rotation, where some of our paramedic participants undertook a six-week rotation through general practice and therefore were not available for pre-hospital patient recruitment. While this affected patient recruitment, it also had the potential to positively impact paramedic interpretation of POCT blood results.

### Limitations

As previously noted, a major limitation of this feasibility study was the small number of patients recruited, which was related to the decision to exclude patients without mental capacity. While this may indicate the potential value of use in this patient population, the opportunity to obtain specific data was significantly reduced. Similarly, our paramedic participant numbers were affected by staff resignations and primary care rotations, which also affected patient recruitment. This feasibility study did not seek to understand the views of those paramedics who were invited to participate but chose not to do so, nor did it seek to understand the impact on other professionals, such as those GPs who were contacted for clinical discussion.

While the paramedic participants were trained in POCT use and provided ongoing support, no observations in practice were undertaken to understand adherence to quality and test procedures, which may have the potential to affect experiences and outcomes.

## Conclusion

The findings of this study suggest that a larger trial is feasible to determine the practicality and utility of using POCT devices to further understand which patient presentations may benefit most and to understand the challenges in urgent care specialist paramedic recruitment and retention. Moreover, a multi-site randomised controlled trial would be useful for understanding the impact on patient outcomes and safety in device application.

Given the cost associated with device use, an economic evaluation is also needed to determine the value of POCT utilisation in the pre-hospital urgent and emergency care environment.

## Acknowledgements

Dr Jon Dickson (GP; University of Sheffield) provided advice on the protocol from the perspective of primary and community care. Dr John Wilson (University of Sheffield) and Dr Shammi Ramlakhan (Sheffield Children’s NHS Foundation Trust) undertook a peer review feedback process of the protocol. The protocol and study proposal were presented to the Sheffield Emergency Care Forum, which comprises patients and public and healthcare professionals for comments and feedback.

## Authors’ contributions

AH and BL were responsible for the conception and design of the study, obtained the research approvals, arranged collaboration agreements and were lead authors of the manuscript. AH acquired funding for the project, and BL designed and delivered educational materials. FB contributed to the design of the study and provided advice and support. RP developed the eCRF, provided data collection and analysis and provided advice on ongoing methodology. BS and AW provided expert guidance and critical review, and AW also provided clinical advice. PB designed standard operating procedures (SOPs) and educational materials for delivery, and KH provided research guidance and collaborated with PB in the design of SOPs. All the authors read and approved the final manuscript. AH acts as the guarantor for this article.

## Conflict of interest

None declared.

## Ethics

This study received approval from the Health Research Authority (HRA) IRAS Project ID: 289975 and is registered with ClinicalTrials.gov (Identifier: NCT05054049). The study protocol was reviewed and approved by the Newcastle and North Tyneside Research Ethics Committee (REC reference: 22/NE/0135). Both the paramedic participants and the patient participants were provided with written information before providing informed written consent. Paramedic participants were able to withdraw their consent at any time, and patient participants could withdraw their consent within seven days of enrolling in the study before their data were anonymised.

## Funding

This study was funded by the NIHR Yorkshire and Humber Patient Safety Translational Research Centre, which did not have the opportunity to comment on this manuscript. Abbott Point of Care, Inc. provided two POCT i-STAT Alinity devices on a loan agreement basis and supported the specialist paramedic training day.
